# Time Frame Affects Vantage Point in Episodic and Semantic Autobiographical Memory: Evidence from Response Latencies

**DOI:** 10.3389/fpsyg.2017.00615

**Published:** 2017-04-20

**Authors:** Jerzy J. Karylowski, Blazej Mrozinski

**Affiliations:** ^1^Institute of Psychology, Polish Academy of SciencesWarsaw, Poland; ^2^Department of Psychology, University of North Florida, JacksonvilleFL, USA; ^3^SWPS University of Social Sciences and HumanitiesWarsaw, Poland

**Keywords:** autobiographical memory, semantic memory, episodic memory, temporal distance, self-judgments, vantage point, accessibility

## Abstract

Previous research suggests that, with the passage of time, representations of self in episodic memory become less dependent on their initial (internal) vantage point and shift toward an external perspective that is normally characteristic of how other people are represented. The present experiment examined this phenomenon in both episodic and semantic autobiographical memory using latency of self-judgments as a measure of accessibility of the internal vs. the external perspective. Results confirmed that in the case of representations of the self retrieved from recent autobiographical memories, trait-judgments regarding unobservable self-aspects (internal perspective) were faster than trait judgments regarding observable self-aspects (external perspective). Yet, in the case of self-representations retrieved from memories of a more distant past, judgments regarding observable self-aspects were faster. Those results occurred for both self-representations retrieved from episodic memory and for representations retrieved from the semantic memory. In addition, regardless of the effect of time, greater accessibility of unobservable (vs. observable) self-aspects was associated with the episodic rather than semantic autobiographical memory. Those results were modified by neither declared trait’s self-descriptiveness (*yes* vs. *no* responses) nor by its desirability (highly desirable vs. moderately desirable traits). Implications for compatibility between how self and others are represented and for the role of self in social perception are discussed.

## Introduction

No sense of personal identity and continuity of the self could exist without memories involving one’s past (Wilson and Ross, 2003). Such autobiographical memories may differ with respect to their event-specificity. On one end of the spectrum we have highly event-specific episodic memories that are immersed in rich sensory details (myself having a dinner last night, myself learning about 9/11 attacks, myself during the 1st day of school, etc.). The other end of the spectrum is occupied by semantic autobiographical memories that are highly generalized, and abstract; they may include representations of self in various social roles (myself as a son, as a student, as a father, as a college professor, etc.), in various periods of time (myself as a child, myself as a teen, myself as a young adult, myself in middle age, myself in retirement, etc.), or even of myself “in general” (Tulving, 1983, 2002; Klein et al., 1992; Conway and Pleydell-Pearce, 2000; Williams et al., 2008; Klein and Loftus, 2014).

The cognitive representations of self emerging as a common threat embodied in our autobiographical memories, regardless of their situation-specificity, are both multi-faceted and dynamic. They become activated – or constructed – depending on chronic accessibility and situational cues (Kihlstrom et al., 2003; Conway, 2005; McConnell, 2011; Skowronski, 2012; McConnell et al., 2013). In addition to specificity, such self-representations can differ in a variety of other ways, including content, predominant modality (verbal, visual, visceral, etc.), evaluative/affective tone, and perspective (internal vs. external).

With regard to internal vs. external perspective, it is not impossible or uncommon for people to consider themselves and their characteristics (e.g., how they talk, look, act) from an external observer’s perspective (Duval and Wicklund, 1972; Carver and Scheier, 1983). However, it is more typical for individuals to give more attention to their own perspectives of themselves. For instance, research shows that, compared to our descriptions of other people, self-descriptions tend to include more privileged, unobservable characteristics – e.g., a person’s internal state such as feeling of joy or shame – that are evident to the individual but more difficult for an external observer to ascertain (McGuire and McGuire, 1988; Prentice, 1990; Andersen et al., 1998; Vazire and Mehl, 2008; Vazire, 2010). A similar pattern of results has been demonstrated for accessibility of unobservable and observable aspects of self-descriptions (Karylowski and Ranieri, 2006; Mrozinski and Karylowski, 2011; Karylowski and Mrozinski, 2017). Specifically, making self-judgments on trait-labels preceded by a verb referring to an internal perspective, such as *feel* (e.g., feels happy, feels sophisticated, etc.) is, in general, faster than making self-judgments on the same trait-labels proceeded by a verb referring to an external perspective such as *look*, or *act* (e.g., looks happy, looks sophisticated, etc.,). Yet, for judgments about others, the opposite pattern is observed with faster judgments on trait-labels preceded by a verb referring to an external perspective (Karylowski and Ranieri, 2006; Mrozinski and Karylowski, 2011).

Those results are not surprising. After all, individuals have more direct access to their own thoughts, plans, feelings, desires, and other features that are not directly observable from the outside than to such unobservable features of others. On the other hand, observable characteristics, including overt behavior, are associated with more direct access when they are displayed by others (cf. Jones and Nisbett, 1972).

Because autobiographical memories not only play a role in preserving one’s self-identity but may also serve as crucial ingredients in forming mental representations of others, both highly familiar and unfamiliar (Smith and Collins, 2009; Gaesser, 2012; Ciramelli et al., 2013; Spreng, 2013), the problem of incompatibility between how self and others are normally represented would have to be resolved. Without such resolution incompatibility between the mental representations of self and mental representations of others would be likely to hinder one’s ability to use self as a guide or a point of comparison in making judgments about other people (Karylowski et al., 2000; Karylowski and Ranieri, 2006). It could also result in biased comparative self-other judgments because different definitions of the same characteristics (more internally based in the case of the perceived self vs. more externally based in the case of the perceived other) would be used when considering one’s own standing vs. the other person’s standing (Niewiarowski and Karylowski, 2008, 2015).

However, the predominance of the internal perspective in content and accessibility of self-representations (and, presumably, the resulting incompatibility between how self and others are represented) is not a universal feature of such representations. Specifically, it does not occur, or is less pronounced, in autobiographical memories when the self is represented in the context of events that occurred in a relatively distant past, i.e., years rather than days (Nigro and Neisser, 1983; McIsaac and Eich, 2002; Piolino et al., 2002; Talarico et al., 2004; Berntsen and Rubin, 2006; Pronin and Ross, 2006; Rice and Rubin, 2009; Sutin and Robins, 2010; Karylowski and Mrozinski, 2017). This effect of time of the event on how the self is represented in memory appears to be robust; it has been reported for different age groups and for both pleasant and unpleasant memories, thus suggesting that with the passage of time self-representations lose their position of an insider and become more compatible with how others are typically represented.

Yet, the evidence for the effect of time of the event on how self is represented in autobiographical memories is based almost entirely on purely introspective measures, i.e., self-reports regarding visual images that participants formed in their minds while visualizing autobiographical events from recent and distant past. The only exception is an experiment by Karylowski and Mrozinski (2017) in which performance-based measures of accessibility of observable and unobservable aspects of self-representation were collected for both recent and distant episodic autobiographical memories. Results showed that self-judgments regarding unobservable characteristics were faster than self-judgments regarding observable characteristics for recent but not for distant episodic autobiographical memories. Moreover, self-judgments regarding unobservable characteristics were faster for memories of recent, compared to memories of distant events. Yet, the opposite emerged in the case of self-judgments regarding observable characteristics – such judgments were actually faster for memories of distant vs. recent events. Thus the experiment provided evidence for the effect of time on how self is represented in memory using a performance-based measure of accessibility.

However, not only is this evidence based on just a single experiment, it is also limited to the episodic autobiographical memory. This last consideration is important because, compared to episodic memory, semantic autobiographical memory has a far greater potential for being accessed across a variety of social situations. Because, by definition, episodic memory is highly event-specific, its cross-situational applicability is hampered (Tulving, 1983, 2002; Conway and Pleydell-Pearce, 2000; Conway, 2005).

One goal of the present experiment was to replicate the effect of time on accessibility of observable and unobservable self-aspects in episodic (event-specific) memory. More importantly, we also attempted to provide evidence for that effect in the domain of semantic (generalized) autobiographical memory, thus extending previous findings. The third, and final goal of the experiment was to address the possibility that the usual predominance of internal perspective as demonstrated by greater accessibility of unobservable compared to observable characteristics will be less pronounced in semantic autobiographical memory than in the episodic autobiographical memory. This last prediction was based on the notion that because semantic memory is more abstract, representations of self in semantic memory will reflect the original (internal) experiential perspective to a lesser degree than the event-specific representations of self encoded in episodic memory (Conway and Pleydell-Pearce, 2000; Conway, 2005).

## Materials and Methods

### Participants

Ninety-six Polish undergraduates (74 women and 22 men, average age *M* = 22.47 years, *SD* = 1.60) participated in the experiment as an option for satisfying an academic extra-curricular activity requirement. To help fully preserve privacy and because the experiment was considered a low risk study, no written consent forms were signed. Instead a formal oral consent was obtained from each participant individually prior to the experiment. The Psychology Research Ethics Committee at the University of Social Science and Humanities approved the study, including the consent procedure.

### Design and Procedure

The experiment was conducted individually in the computer lab. Half of the participants (*N* = 48) were assigned to the episodic memory condition and the other half to the semantic memory condition. For the episodic memory condition, the procedure was modeled on the procedure in Karylowski and Mrozinski (2017). Participants in that condition were asked to make judgments regarding how they were feeling and how they acted in two *social situations* from their past. Participants were given an example of a family dinner as a type of a social situation they could choose. In contrast, participants in the semantic memory condition, were asked to make judgments regarding how they were typically feeling and how they typically acted in two periods of their lives. Thus, the two between-participant conditions differed with respect to the degree to which self-judgments involved episodic (event-specific) vs. semantic (generalized) autobiographical memories.

For all participants, the experimental task was divided into two parts, one involving “recent past” and the other one involving “distant past” (defined as “about 10 years ago”). The order of the two parts was counterbalanced across participants. At the onset of each part, participants were asked to try to vividly recall either the event in which they participated (episodic memory condition) or their “view of yourself” (semantic memory condition), either recently or in a distant past, and to write – on a provided sheet of paper – what came to their minds. This was done to ensure that participants accessed event-specific (episodic memory condition) or general (semantic memory condition) autobiographical memories regarding recent and distant past.

For each of the two parts, recalling and describing autobiographical memories was followed by two blocks of self-ascription judgments with 20 judgments per block. Thus each participant made a total of 80 judgments. Each judgment involved a different personal characteristic. The characteristics ranged from neutral/ambivalent (e.g., conciliatory, critical, humble, and obedient) to highly positive (e.g., ambitious, friendly, loyal, and smart)^1^. The order of adjectives was non-systematic and constant for all participants.

Within each of the two parts (referring to recent vs. remote autobiographical memory), characteristics in one block of 20 judgments were always preceded by a qualifier *feel* (e.g., felt conciliatory, felt critical, felt humble, felt obedient, felt ambitious, felt friendly, felt loyal, and felt smart) and in the other block by a qualifier *act* (e.g., acted conciliatory, acted critical, acted humble, acted obedient, acted ambitious, acted friendly, acted loyal, and acted smart), referring to either internal (unobservable) or external (observable) manifestation of a given characteristic. The order of *feel* vs. *act* blocks was counterbalanced across participants. Thus, with the order of adjectives constant, each adjective was used for each of the four combinations of judgments (feel vs. act judgment regarding recent vs. distant event) for exactly 25% of participants. The resulting design was a 2 (episodic vs. semantic memory) × 2 (feel vs. act judgment) × 2 (recent vs. distant event) mixed-model with the first variable manipulated between- and the remaining two variables manipulated within-participants (and counterbalanced).

Participants were asked to work at the fastest comfortable pace and were informed that both their responses and their response latencies were recorded. Responses were provided on a two-point *(Yes/No)* scale using “A” and “L” keys on a standard keyboard with the assignment of the two keys counterbalanced across participants. The use of a two-point scale constituted a departure from how accessibility was measured in the previous experiment (Karylowski and Mrozinski, 2017) which employed a five-point Likert-type scale. While the five-point scale has an advantage of providing a more precise measure of the degree of participants’ endorsement of a given item, its use in assessing accessibility is controversial (cf., Fazio, 1990). This is mainly because using a non-dichotomous scale increases noise variance due to the motoric search time to find relevant key to press.

## Results

### Descriptions of Recent and Distant Memories

Descriptions of recent and distant memories in the episodic memory and semantic memory conditions, were analyzed to examine possible differences between the four sets with respect to length of the description (number of words), specificity (event-specific, categorical, extended, or semantic), and overall valence (rather positive, neutral/undecided, or rather negative)^2^. A 2 (memory type: episodic vs. semantic) × 2 (time: recent vs. distant) mixed model analysis of variance (ANOVA) was performed on length of the descriptions. This analysis revealed a significant effect of the memory type with longer descriptions in the episodic memory, *M* = 64.97 words, compared to the semantic memory condition, *M* = 38.41 words, *F*(1,94) = 23.25, *p* < 0.001, η^2^ = 0.20, a result consistent with a more detailed content of episodic memories. Neither the main effect of time, nor the interaction approached significance, both *p*s > 0.2.

Event-specificity of each description was assessed using a scoring system developed by Raes et al. (2007). Each memory was classified as either event-specific or as an instance of (over)general memory – either categoric memory, extended memory, or semantic associate. Using those categories, in the episodic memory condition, 96 out of the 96 descriptions were classified as specific (e.g., …this was a party for my sister’s 24th birthday..…). In contrast, in the semantic memory condition 94 out of 96 descriptions were classified as semantic associates (e.g., …I used to be a very outgoing person….) and the remaining two, one in each time category, were classified as instances of extended memory (e.g., …during my 1st year of college I was very busy..…). Thus, consistently with the experimental manipulation, participants in the episodic memory condition produced descriptions that, without exception, were event-specific while participants in the semantic memory conditions produced descriptions that were overwhelmingly semantic.

For exploratory purposes, descriptions of recent and distant memories in the two experimental conditions were also compared with respect to an overall valence. This was done by assigning each memory to one of three categories: “rather positive,” “neutral or undecided,” and “rather negative.” Overall, out of 192 descriptions, 140 (72.92%) were classified as rather positive, 36 (18.75%) as neutral or undecided, and 16 (8.33%) as rather negative. No statistically significant differences associated with either the experimental condition (episodic memory vs. semantic memory), time of the event (distant vs. recent), or their interaction were detected, all *p*s > 0.2.

### Percentages of Yes Responses in Self-Judgments

On average, participants responded with *yes* in 68.28% of trials, a percentage significantly higher than the 50% expected by chance, *t*(95) = 15.61, *p* < 0.001. This is not surprising, given that characteristics used in self-judgments were, on average, positive and no negative characteristics were included. Thus, the predominance of the yes responses would be consistent with motivation to preserve and enhance positive self-esteem. Operation of such self-esteem motive in autobiographical memory, while outside of the main focus of the current paper, has been well established in the literature (Alicke and Sedikides, 2011).

A 2 (memory type: episodic vs. semantic) × 2 (judgment type: feel vs. act) × 2 (time: recent vs. distant) mixed model ANOVA conducted on the average percentages of the *yes* responses revealed the main effect of time with the higher percentage of such responses for recent, compared to distant autobiographical memories, *M* = 73.55% and *M* = 63.04%, respectively *F*(1,94) = 24.90, *p* < 0.001, η^2^ = 0.21. Like the predominance of the *yes* responses overall, this result is consistent with operation of the self-esteem motive. Positive self-views related to recent rather than distant past, are likely to lead to the enhanced self-esteem due to an increased sense of successfully overcoming obstacles and improving over-time (Wilson and Ross, 2003). No other significant effects emerged.

### Response Latencies of Self-Judgments

Response latencies of self-judgments involving unobservable (feel) and observable (act) characteristics constituted the principal dependent variable of interest. The main analysis was conducted for latencies regardless of whether participant’s response was *yes* or *no*. This was based on the assumption that both *yes* and *no* responses constitute self-judgments and that the *no* response may be interpreted as endorsements of a characteristic opposite to the one on which the self-judgments are made. Moreover, conducting the main analysis of latency data for *yes* and *no* responses together helps to ensure the integrity of the counterbalancing employed in the design (obviously, no counterbalancing scheme could anticipate self-judgments of individual participants). Nevertheless, effects associated with the response type, *yes* vs. *no*, were assessed in auxiliary analyses reported after results of the main analysis are presented.

Latencies shorter than 500 ms (0.4% of responses) and latencies longer than 10,000 ms (0.6% of responses) were considered invalid and were dropped from the analysis. Also, to further reduce positive skew, latencies were converted to natural logarithms (see Winer, 1971, p. 400). Preliminary analysis revealed a practice effect across the 80 trials, with higher serial position associated with shorter latencies – the average Fisher’s *z-*values were *M* = -0.26 in the episodic memory condition and *M* = -0.23 in the semantic memory condition, corresponding to *r* = -0.25 and *r* = -0.23, respectively. The means were reliably different from 0; *t*(47) = 13.08, *p* < 0.001, η^2^ = 0.20 in the episodic memory condition and *t*(47) = 10.43, *p* < 0.001, η^2^ = 0.16 in the semantic memory condition. Accordingly, in order to reduce error variance and to increase power, the main analysis was performed on latencies regression-adjusted for the effect of serial position.

**FIGURE 1:**
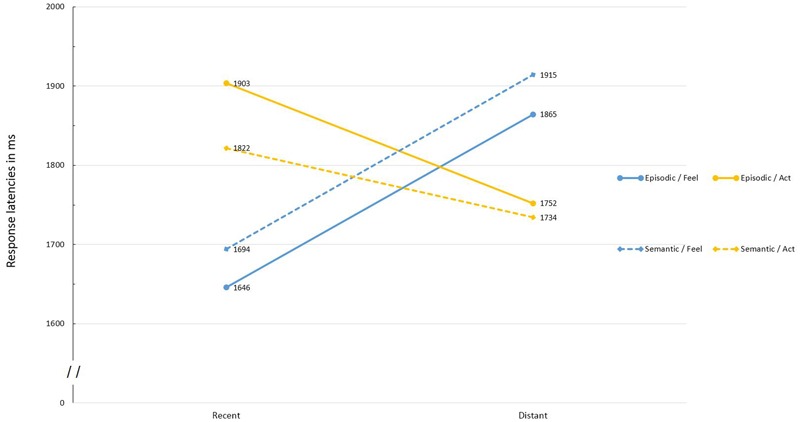
**Response latencies for *feel* and *act* self-judgments involving recent and distant, episodic and semantic autobiographical memories**.

A 2 (memory type: episodic vs. semantic) × 2 (judgment type: feel vs. act) × 2 (time: recent vs. distant) mixed model ANOVA was conducted on latencies and the means are presented in **Figure 1**.^3^ The main effect of time was significant, *F*(1,94) = 12.98, *p* < 0.001, η^2^ = 0.12. Specifically, judgments regarding recent memories were, on average, faster, *M* = 1766 ms, than judgments regarding distant autobiographical memories, *M* = 1816 ms This effect was qualified by the predicted Time × Judgment Type interaction, *F*(1,94) = 12.73, *p* < 0.001, η^2^ = 0.12. Comparison of simple effects revealed that for recent memories, the feel judgments were faster, *M* = 1670 ms, than the act judgments, *M* = 1863 ms, *F*(1,94) = 12.58, *p* = 0.001, η^2^ = 0.12. However, for the distant memories, the act judgments were faster, *M* = 1743 ms, than the feel judgments, *M* = 1890 ms, *F*(1,94) = 7.64, *p* = 0.007, η^2^ = 0.08. In addition, the feel judgments were faster for the recent, compared to the distant memories, *F*(1,94) = 20.08, *p* < 0.001, η^2^ = 0.18. Yet the act judgments, were faster for the distant memories, *F*(1,94) = 5.60, *p* < 0.020, η^2^ = 0.06. Importantly, the Time × Judgment Type interaction was not qualified by a three-way interaction involving memory type, *F*(1,94) < 1. In fact, the Time × Judgment Type interaction was significant both in the episodic memory condition, *F*(1,47) = 6.53, *p* = 0.014, η^2^ = 0.12, and in the semantic memory condition, *F*(1,47) = 6.27, *p* = 0.016, η^2^ = 0.12.

Returning to the omnibus ANOVA, the only other effect approaching statistical significance was the interaction between memory type and judgment type, *F*(1,94) = 3.92, *p* = 0.051, η^2^ = 0.04. Specifically, for the feel judgments, self-judgments based on the content retrieved from the episodic memory, were significantly faster, *M* = 1755 ms, than those based on the content retrieved from the semantic memory, *M* = 1804 ms, *F*(1,94) = 3.98, *p* = 0.049, η^2^ = 0.04. The opposite pattern was observed for the act judgments, *M* = 1828 ms and *M* = 1778 ms, in the episodic memory condition and the semantic memory condition, respectively, *F*(1,94) = 3.86, *p* = 0.052, η^2^ = 0.04. In addition, for self-judgments based on the episodic memory, the feel judgments were significantly faster than the act judgments, *F*(1,47) = 4.98, *p* = 0.030, η^2^ = 0.10. This, however, was not the case for self-judgments based on the semantic autobiographical memory, *F*(1,47) < 1. Thus, it appears, that the accessibility of observable (act) as compared to unobservable (feel) aspects of autobiographical memories is facilitated both by retrieving older autobiographical memories and by retrieving memories that are semantic rather than event-specific. Furthermore, our data suggest that the two factors operate independently (no three-way interaction).

### Auxiliary Analyses

Auxiliary analyses were conducted to assess the robustness of the main findings vis-a-vis two additional factors not included in the main analysis of the latency data: response type (*yes* vs. *no*) and favorability of the characteristic for which judgment was made (moderately vs. highly favorable).

#### Response Type

As stated earlier, in order to ensure the integrity of the counterbalancing employed in the design, for the main analysis, the latencies were analyzed without distinguishing between *yes* and *no* responses. Yet, arguably, pooling both kinds of response latencies together makes it difficult to distinguish between effects that might have been due to differences between judgment categories in terms of the percentages of *yes* and *no* responses, on one hand, and the genuine accessibility effects, on the other. This is because compared to the *yes* responses, the *no* responses are typically slower (Luce, 1986; Fazio, 1990).

It could be argued that while including response type in the analysis should result in effects of the response type variable consistent with the operation of the self-esteem motive, both the Judgment Type × Time interaction and the Judgment Type × Memory Type interaction reported in the main analysis should remain intact. Moreover, if the assumption that the *no* responses may be interpreted as endorsements of characteristics opposite to those explicitly included in the judgment task is valid, those interactions should not be qualified by three-way interactions with the response type. This is because, there is no theoretical reason why those two-way interactions should occur for favorable characteristics but not for their unfavorable opposites.

To check those predictions, a 2 (memory type: episodic vs. semantic) × 2 (judgment type: feel vs. act) × 2 (time: recent vs. distant) × 2 (response: *yes* vs. *no*) mixed model ANOVA was conducted on response latencies. This analysis confirmed both the Time × Judgment Type interaction and the Memory Type × Judgment Type interaction, *F*(1,94) = 10,62, *p* = 0.002, η^2^ = 0.10 and *F*(1,94) = 4.51, *p* = 0.036, η^2^ = 0.05, respectively. Moreover, neither of those interactions was qualified by a higher order interaction, all *p*s > 0.2, an indication that neither of the predicted two-way interactions was contingent upon the specific levels of the remaining variables, including the response type.

Of secondary interest, there was an unsurprising (see Luce, 1986; Fazio, 1990) strong main effect of the response type, *F*(1,94) = 89.19, *p* < 0.001, η^2^ = 0.49, with faster *yes, M* = 1729 ms, compared to the *no* responses, *M* = 2110 ms. A two-way interaction between time and response type was also significant, *F*(1,94) = 8.22, *p* = 0.005, η^2^ = 0.08. *Post hoc* comparisons showed a pattern consistent with operation of the self-esteem motive. Specifically, the *yes* responses (endorsing positive self-characteristics) were faster for judgments involving recent, *M* = 1694 ms, than for judgments involving distant past, *M* = 1763 ms, *F*(1,94) = 8.55, *p* < 0.004, η^2^ = 0.08. However, the *no* responses were actually faster for judgments involving distant past, *M* = 2038 ms, than for those involving recent past, *M* = 2183 ms, *F*(1,94) = 4.88, *p* < 0.030, η^2^ = 0.05. In addition, although the *yes* responses were faster both for judgments involving recent and for judgments involving distant past, consistently with the self-esteem motive interpretation, the effect was larger for the recent memory, *F*(1,94) = 72.82, *p* < 0.001, η^2^ = 0.44, than for the distant memory, *F*(1,94) = 28.64, *p* < 0.001, η^2^ = 0.23.

#### Favorability

An analogous auxiliary analysis was conducted to examine accessibility effects of memory type, time, and judgment type depending on the favorability of the characteristic involved in the judgment. Specifically, the 80 characteristics were split by median of their favorability ratings as reported by Wojciszke (2010, Appendix A). Because no unfavorable characteristics were used in the present experiment, the median split reflects the distinction between moderately favorable vs. highly favorable characteristics. A 2 (memory type: episodic vs. semantic) × 2 (judgment type: feel vs. act) × 2 (time: recent vs. distant) × 2 (favorability: moderate vs. high) mixed model ANOVA, once again, confirmed both the Time × Modality interaction, *F*(1,94) = 11.41, *p* = 0.001, η^2^ = 0.11, and the Memory Type × Modality interaction, *F*(1,94) = 4.80, *p* = 0.031, η^2^ = 0.05. Moreover, neither of those two two-way interactions was modified by a higher order interaction, all *p*s > 0.2. Thus, neither of the two crucial interactions appears to be contingent upon whether highly favorable or just moderately favorable characteristics were used in self-judgments (or on the level of any other variable included in the analysis).

Of a lesser relevance, there was a strong main effect of favorability, *F*(1,94) = 51.28, *p* < 0.001, η^2^ = 0.35. Once again, consistently with operation of the self-esteem motive, judgments involving highly favorable characteristics were faster, *M* = 1720 ms, than judgments involving moderately favorable characteristics, *M* = 1824 ms. Moreover, the interaction between favorability and memory type was also significant, *F*(1,94) = 10.72, *p* = 0.001, η^2^ = 0.10. Follow up tests showed that, in the case of highly favorable characteristics, self-judgments were faster for the semantic memory condition, *M* = 1696 ms, then for the episodic memory condition, *M* = 1744 ms, *F*(1,94) = 8.37, *p* = 0.005, η^2^ = 0.08. However, in the case of characteristics that were only moderately favorable, self-judgments were faster for the episodic memory condition, *M* = 1800 ms, then for the semantic memory condition, *M* = 1848 ms, *F*(1,94) = 11.06, *p* = 0.001, η^2^ = 0.11. In addition, although judgments involving highly favorable characteristics were faster for both memory types, the effect was larger in the semantic, *F*(1,47) = 52.40, *p* < 0.001, η^2^ = 0.53 then in the episodic memory condition, *F*(1,47) = 7.86, *p* = 0.007, η^2^ = 0.14.

## Discussion

Results of the present experiment show that the relative accessibility of observable vs. unobservable trait-aspects in autobiographical memories varies depending on whether the memory involves recent or a more distant past. In the case of representations of the self retrieved from recent autobiographical memories, trait-judgments regarding unobservable (covert) self-aspects are faster than trait judgments regarding observable (overt) self-aspects, indicating greater accessibility of unobservable (covert) self-aspects. Yet, in the case of self-representations retrieved from memories of a more distant past, judgments regarding observable (overt) self-aspects are faster, indicating greater accessibility of observable (overt) self-aspects. Thus, with the passage of time, self-representations embedded in personal memories appear to lose their distinct reliance on the internal perspective and to assume a more external perspective. This suggests that such older self-representations with their greater emphasis on observable aspects are more compatible with how other people are typically represented (e.g., McGuire and McGuire, 1988; Prentice, 1990; Karylowski and Ranieri, 2006; Vazire and Mehl, 2008; Vazire, 2010).

Our results show that the effect of time (recent vs. distant memories) on accessibility of observable and unobservable self-aspects occurs both for the event-specific (episodic) and for the generalized (semantic) autobiographic memories. For the event-specific memory, this replicates a previous finding (Karylowski and Mrozinski, 2017). The replication is noteworthy not only because previous empirical support was based on just a single experiment but also because the current experiment utilized a more established experimental task, with a dichotomous *yes/no* scale, rather than a 5-point Likert-type scale (see Fazio on the controversies regarding using Likert-type response scales in accessing accessibility).

The finding that the effect of time is not limited to the representation of self in episodic memory but extends to how self is represented in semantic memory is not trivial. Situation-specificity of episodic self-representations is likely to make such representations less relevant across a wide spectrum of social situations involving others. If so, increased compatibility between how others are typically represented (primarily in terms of observable features) and how self in the distant past is represented, may be insufficient to overcome lack of compatibility resulting from high situation-specificity of the representations. In contrast, highly generalized (semantic) self-representations should be more likely to appear as good candidates for the recruitment as points of reference in making judgments about others. The present experiment suggests that this would be the case especially for generalized representations of the self associated with the relatively distant, rather than with the recent past. This prediction should be explored in future research.

Our results also show that, regardless of the effect of time, greater accessibility of observable (vs. unobservable) self-aspects is associated with the semantic rather than episodic autobiographical memory. While, to our knowledge, such finding has not been previously reported, it is not surprising. It is fully consistent with the notion of the secondary nature of semantic autobiographical memory that, compared to its episodic counterpart, is more abstract, more integrated with general social knowledge and thus less dependent on the subjective, experiential perspective predominant at encoding (Conway and Pleydell-Pearce, 2000; Conway, 2005).

All those predicted effects appear to be robust both with respect the response type (yes vs. no responses) and with respect to favorability (highly favorable vs. moderately favorable characteristics) – no higher order interactions with either the response type or favorability emerged. Yet, it should be noted that the effects, while significant, were not particularly strong. This was in contrast to more powerful effects that were consistent with the operation of the self-esteem motive but were not directly related to the goals of the present experiment. While such motivational effects are probably unavoidable in any research involving self-judgments, because they tend to be fairly strong, they should always be carefully considered, at every stage even when, like in the case of the present experiment, they are not directly relevant to the research question(s). This must include sufficient power to guard against type-1 error in testing effects that might be highly theoretically relevant but empirically more subtle than effects associated with the self-esteem motive.

## Conclusion

Results of the present experiment confirmed that in the case of recent autobiographical memories, trait-judgments regarding unobservable (privileged) aspects of self-knowledge were more cognitively accessible than trait judgments regarding observable (overt) aspects. Yet, in the case of autobiographical memories from a more distant past, judgments regarding observable (overt) self-aspects were more cognitively accessible. Those findings occurred for both episodic and semantic autobiographical memories, for both highly desirable and moderately desirable characteristics, and for both self-descriptive and non-self-descriptive characteristics. In addition, overall, accessibility advantage of unobservable aspects of self-knowledge was greater for episodic compared to semantic memories.

## Ethics Statement

This study was carried out in accordance with the recommendations of ‘SWPS University of Social Sciences and Humanities Departmental Ethics Committee’ with oral informed consent from all subjects. All subjects gave oral informed consent in accordance with the Declaration of Helsinki. The protocol was approved by the ‘SWPS University of Social Sciences and Humanities Departmental Ethics Committee.’ The use of oral consent was approved in order to safeguard anonymity.

## Author Contributions

Both authors contributed substantially at every stage. See below for listing of approximate percentage contribution by stage. Conception and Design: JK 60% and BM 40%. Implementation: JK 20% and BM 80%. Data analysis: JK 40% and BM 60%. Writing: JK: 60% and BM 40%.

## Conflict of Interest Statement

The authors declare that the research was conducted in the absence of any commercial or financial relationships that could be construed as a potential conflict of interest.

## Footnotes

1 Favorability ratings were based on norms reported by Wojciszke (2015, Appendix A).

2 Both event-specificity and valence were rated by BM who was blind with respect to the semantic memory vs. episodic memory experimental condition. Satisfactory levels of inter-rater agreement (97 and 86% for event-specificity and valence, respectively) were established using a separate sample of 88 episodic memory protocols with JJK serving as the remaining rater (Karylowski and Mrozinski, 2017, footnotes 3 and 4).

3 To facilitate presentation, throughout the paper, mean latencies are presented after being converted back to milliseconds.
